# Lipoteichoic Acid Is Involved in the Ability of the Immunobiotic Strain *Lactobacillus plantarum* CRL1506 to Modulate the Intestinal Antiviral Innate Immunity Triggered by TLR3 Activation

**DOI:** 10.3389/fimmu.2020.00571

**Published:** 2020-04-09

**Authors:** Hiroya Mizuno, Lorena Arce, Kae Tomotsune, Leonardo Albarracin, Ryutaro Funabashi, Daniela Vera, Md. Aminul Islam, Maria Guadalupe Vizoso-Pinto, Hideki Takahashi, Yasuko Sasaki, Haruki Kitazawa, Julio Villena

**Affiliations:** ^1^Food and Feed Immunology Group, Laboratory of Animal Products Chemistry, Graduate School of Agricultural Science, Tohoku University, Sendai, Japan; ^2^Infection Biology Laboratory, Instituto Superior de Investigaciones Biológicas (INSIBIO), CONICET-UNT, Tucumán, Argentina; ^3^Livestock Immunology Unit, International Education and Research Center for Food Agricultural Immunology, Graduate School of Agricultural Science, Tohoku University, Sendai, Japan; ^4^Laboratory of Immunobiotechnology, Reference Centre for Lactobacilli (CERELA-CONICET), Tucuman, Argentina; ^5^Laboratorio de Ciencias Básicas Or. Genética, Facultad de Medicina de la Universidad Nacional de Tucuman, Tucumán, Argentina; ^6^Department of Medicine, Faculty of Veterinary Science, Bangladesh Agricultural University, Mymensingh, Bangladesh; ^7^Plant Immunology Unit, International Education and Research Center for Food Agricultural Immunology, Graduate School of Agricultural Science, Tohoku University, Sendai, Japan; ^8^Graduate School of Agriculture, Meiji University, Kawasaki, Japan

**Keywords:** immunobiotic, *D-alanyl-lipoteichoic acid biosynthesis protein* mutant, *Lactobacillus plantarum* CRL1506, intestinal immunity, lipoteichoic acid

## Abstract

Studies have demonstrated that lipoteichoic acid (LTA) is involved in the immunomodulatory properties of some immunobiotic lactobacilli. The aim of this work was to evaluate whether LTA contributes to the capacity of *Lactobacillus plantarum* CRL1506 in modulating the intestinal innate antiviral immune response. A D-alanyl-lipoteichoic acid biosynthesis protein (*dltD*) knockout CRL1506 strain (*L. plantarum*Δ*dltD*) was obtained, and its ability to modulate Toll-like receptor (TLR)-3-mediated immune response was evaluated *in vitro* in porcine intestinal epithelial (PIE) cells and *in vivo* in Balb/c mice. Wild-type (WT) CRL1506 (*L. plantarum* WT) was used as positive control. The challenge of PIE cells with the TLR3 agonist poly(I:C) significantly increased interferon (IFN)-β, interleukin (IL)-6, and monocyte chemoattractant protein (MCP)-1 expressions. PIE cells pretreated with *L. plantarum*Δ*dltD* or *L. plantarum* WT showed higher levels of IFN-β while only *L. plantarum* WT significantly reduced the expression of IL-6 and MCP-1 when compared with poly(I:C)-treated control cells. The oral administration of *L. plantarum* WT to mice prior the intraperitoneal injection of poly(I:C) significantly increased IFN-β and IL-10 and reduced intraepithelial lymphocytes (CD3^+^NK1.1^+^CD8αα^+^) and pro-inflammatory mediators (TNF-α, IL-6, and IL-15) in the intestinal mucosa. Similar to the WT strain, *L. plantarum*Δ*dltD*-treated mice showed enhanced levels of IFN-β after poly(I:C) challenge. However, treatment of mice with *L. plantarum*Δ*dltD* was not able to increase IL-10 or reduce CD3^+^NK1.1^+^CD8αα^+^ cells, TNF-α, IL-6, or IL-15 in the intestine. These results indicate that LTA would be a key molecule in the anti-inflammatory effect induced by the CRL1506 strain in the context of TLR3-mediated inflammation.

## Introduction

One of the fundamental problems of the world related to nutrition is the immunosuppression associated with malnutrition (1, 2). Undernutrition is one of the factors that contribute most to the global burden of disease, with increasing numbers affecting the most vulnerable populations in developing countries (2). More than a third of child deaths worldwide are attributed to malnutrition and its profound impact on the host resistance to infections. In this regard, diarrhea remains the leading cause of death of children, particularly in those with severe malnutrition (3). Although in recent years it has been possible to reduce infant mortality and morbidity resulting from rotavirus gastroenteritis, such infection is still a persistent problem worldwide and is one of the most common causes of hospitalization and mortality in children. Globally, at least 200,000 children under 5 years of age die of diarrhea every year due to the severe dehydration and electrolyte disorders caused by rotavirus infection (4). The majority of rotavirus-related deaths (>80%) are found in low-income countries, where there is a high prevalence of child malnutrition and limited access to medical care (5).

The World Health Organization has proposed a cohesive approach to end preventable diarrhea deaths in children (3). The plan proposes interventions to create healthy environments, promotes practices to protect children from infectious diseases, and ensures that all children have access to appropriate preventive and treatment measures. The document emphasizes the importance of healthy food and vaccination for the prevention of intestinal infections in children. In this regard, various clinical trials and animal model studies have demonstrated the ability of probiotic lactic acid bacteria (LAB) with immunomodulatory activities, also known as immunobiotics, to improve the resistance to intestinal viral infections (6–8). Although great advances have been made in the use of immunobiotics for the development of immunomodulatory functional foods or as adjuvants in experimental mucosal vaccines (6–8), deeper studies are still necessary to achieve a better understanding of their interaction with the immune system. Deeper knowledge of the cellular and molecular interactions of immunobiotics with host immune and non-immune cells could help in the selection of the most efficient strains and lay the scientific basis for their safe use, in particular in high-risk populations such as malnourished children.

Previous *in vitro* transcriptomic studies showed that *Lactobacillus plantarum* CRL1506 is able to modulate the expression of type I interferons (IFNs), antiviral factors, cytokines, chemokines, and adhesion molecules in porcine intestinal epithelial (PIE) cells challenged with the Toll-like receptor (TLR)-3 ligand poly(I:C) (9). In addition, studies in porcine antigen-presenting cells isolated from Peyer’s patches demonstrated that the CRL1506 strain differentially modulated the expression of IFN-γ, interleukin (IL)-1β, IL-12, IL-6, and tumor necrosis factor (TNF)-α (10). Moreover, *L. plantarum* CRL1506 was able to activate porcine CD172a^+^CD11R1^*high*^ dendritic cells (DCs) and CD172a^+^CD11R1^–^ macrophages as demonstrated by the enhanced levels of the major histocompatibility complex (MHC)-II and co-stimulatory molecules CD80/86 (10). The patterns of IFNs, cytokines, and chemokines induced by *L. plantarum* CRL1506 in resident intestinal immune and non-immune cells allow us to predict an improved recruitment and activation of immune cells to the gut mucosa, which could beneficially influence the elimination of the virus. On the other hand, taking into consideration that the deregulated activation of immune cells and/or their excessive recruitment into the infected tissue could contribute to the local cellular damage in the context of viral infection, we also evaluated the ability of *L. plantarum* CRL1506 to protect against the TLR3-mediated intestinal inflammatory alterations. Our *in vivo* studies in mice demonstrated that orally administered CRL1506 strain, prior to the intraperitoneal challenge of animals with poly(I:C), significantly reduced the severity of intestinal damage triggered by TLR3 activation. The beneficial effect of the immunobiotic strain was related to its capacity of modulating the expression of IL-15 and retinoic acid early inducible-1 (RAE1) in epithelial cells and the activation of CD3^+^NK1.1^+^CD8αα^+^ intraepithelial lymphocytes (IELs) (11).

In order to gain deeper knowledge of the bacterial molecules involved in the beneficial effects of *L. plantarum* CRL1506, in this work, we aimed to evaluate whether lipoteichoic acid (LTA) contributes to the capacity of the immunobiotic strain of modulating the intestinal innate antiviral immune response. A D-alanyl-lipoteichoic acid biosynthesis protein (*dltD*) knockout CRL1506 strain (*L. plantarum*Δ*dltD*) was obtained, and its ability to modulate TLR3-mediated immune response was evaluated *in vitro* and *in vivo*. Similar to the wild-type (WT) strain, the mutant *L. plantarum*Δ*dltD* was able to differentially modulate IFN-γ and IFN-β in response to poly(I:C) challenge *in vitro* and *in vivo*. However, *L. plantarum*Δ*dltD* was not able to increase IL-10 or reduce the inflammatory damage mediated by CD3^+^NK1.1^+^CD8αα^+^ cells and IL-15 in the intestinal mucosa. The results of this work indicate that LTA would be a key molecule in the anti-inflammatory effect induced by *L. plantarum* CRL1506 in the context of TLR3-mediated inflammation.

## Materials and Methods

### Microorganisms

*Lactobacillus plantarum* CRL1506 was obtained from the Reference Centre for Lactobacilli (CERELA CONICET) Culture Collection (Tucuman, Argentina). Cultures were kept freeze-dried and then rehydrated using the following medium: tryptone, 10.0 g; meat extract, 5.0 g; peptone, 15.0 g; and distilled water, 1 L, pH 7. Bacteria were cultured for 12 h at 37°C (final log phase) in Man–Rogosa–Sharpe broth (MRS, Oxoid, Cambridge, United Kingdom).

**FIGURE 1:**
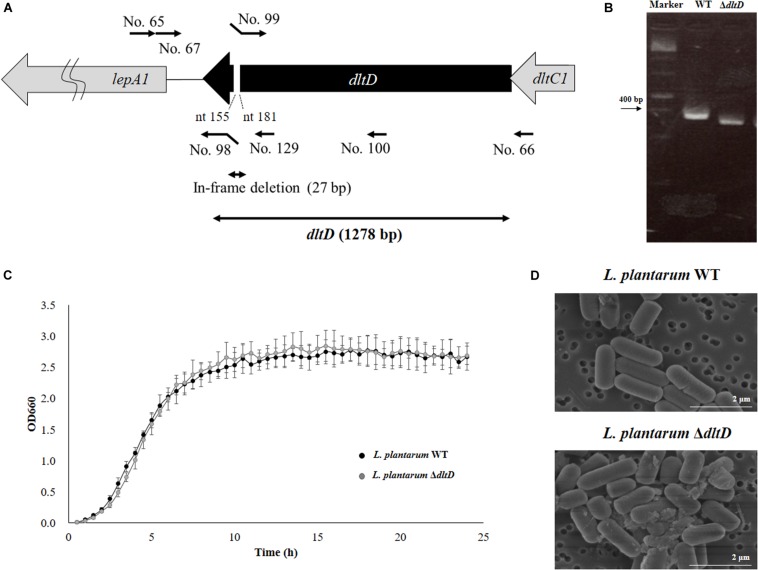
Development of a D-alanyl-lipoteichoic acid biosynthesis protein (*dltD*) mutant from *Lactobacillus plantarum* CRL1506. **(A)** Summary of the strategy used for the development of *L. plantarum* Δ*dltD* by double-crossover. Primer sequences are listed in Table 1. **(B)** Confirmation of the deletion of 27 bp in the *dltD* gene by PCR analysis. **(C)** Growth curve and **(D)** electron microscope analysis of wild-type (WT) and Δ*dltD* mutant from *Lactobacillus rhamnosus* CRL1506.

The mutant *D-alanyl-lipoteichoic acid biosynthesis protein* knockout *L. plantarum* CRL1506 strain (*L. plantarum* Δ*dltD*) was obtained according to the method described by Yamauchi et al. (12). Briefly, the *dltD* gene was obtained by PCR using primers No. 65 and No. 66 (Table 1 and Figure 1). By using this gene fragment as a template, the 27-bp in-frame deletion of *dltD* was obtained by PCR using primers No.67 and No. 98, followed by primers No. 99 and No. 100. These fragments were combined by overlap PCR using No. 67 and No. 100 (Table 1 and Figure 1). This fragment was digested by *Sac*I and *Kpn*I and then inserted into pSG^+^E2 (thermo-sensitive replicon) that was obtained from the Food Research and Development Center, Meiji Dairies Corporation (Tokyo, Japan), by DNA Ligation Kit Ver1 (Takara Bio Inc., Japan) to generate the pSG^+^dD plasmid. The construction of this vector was performed in *Lactococcus lactis* IL1403 (kindly given by the Food Research and Development Center, Meiji Dairies Corporation, Tokyo, Japan). The pSG^+^dD was electroporated into *L. plantarum* CRL1506 according to the method described by De Keersmaecker et al. (13). A double crossover event was performed according to the method described by Biswas et al. (14) in order to obtain a Δ*dltD L. plantarum* CRL1506 strain. The 27-bp deletion in the *dltD* gene of CRL1506 mutant was confirmed by sequencing.

**TABLE 1:** Sequence of the primers used in this study.

**Primer number**	**Sequence**
No. 65	GGACGTTAACCTGTTTGATGAAGGA
No. 66	CCATCCTTTGCTTGATAGTGTAACTCTAC
No. 67	ACACGGTACCGGGTTCCGTCCAACTTTTATTGGAA
No. 98	CATCATCCAGCGCCTCGTTGGCTGACAAGG
No. 99	CCTTGTCAGCCAACGAGGCGCTGGATGATG
No. 100	CACAGAGCTCGCCCGCAATTCCAAAACGTG
No. 129	GATGCATTGGATCAAATCGTG

Scanning electron microscope (SEM) was used to analyze the bacterial surfaces of WT *L. plantarum* CRL1506 (*L. plantarum* WT) and *L. plantarum* Δ*dltD*. For this purpose, the bacteria were cultured for 16 h at 37°C in MRS broth and then centrifuged (6,000 × *g* for 5 min at 5°C), and the pellets were diluted 10-fold with phosphate buffered saline (PBS). Samples were dropped into membrane filter polycarbonate of 0.2 μm × 13 mm (ADVANTEC), and the bacterial cells were placed on the filter using suction filtration. These filters were allowed to stand in 2% glutaraldehyde for 1 h at room temperature to fix the cells. The samples were observed by SEM.

Western blot analysis and high-performance liquid chromatography (HPLC) were performed to analyze LTA in *L. plantarum* WT and *L. plantarum* Δ*dltD*. The collection of cell walls was performed according to the method described by Hirose et al. (15). The overnight cultures of WT and Δ*dltD* in MRS medium were added to fresh MRS medium and cultured at 37°C for 5 h (middle of log phase). The cultures were centrifuged at 6,000 × *g* for 5 min. PBS wash was performed twice, and the pellets were resuspended by PBS. Then, bacterial cells were disrupted by Micro Smash^TM^ MS-100R (Tomy Seiko Co., Ltd., Japan) (4,500 rpm for 3 min on ice). The recovered liquid was centrifuged at 5,000 rpm for 20 min at 4°C, and the supernatant was centrifuged again at 40,000 rpm for 20 min at 4°C. These pellets were freeze dried. For Western blot analysis, anti-LTA antibody (Ab), Mouse Lipoteichoic Acid Monoclonal Antibody (GeneTex, Inc., United States) was used as primary Ab (1:500) for 16 h at 4°C and Alkaline Phosphatase AffiniPure Goat Anti-Mouse IgG (H + L) (Jackson ImmunoResearch Laboratories, Inc., United States) was used as secondary Ab (1:20,000) for 1 h at room temperature. The ECF reaction was performed for 5 min by using ECF Substrate for Western blotting (GE HealthCare, Inc., United States). For HPLC, acid hydrolysis of the samples was performed by the method described Hirose et al. (15). The samples were diluted in 6N hydrochloric acid (HCl) at 100°C for 15 h and were neutralized, added to equal volume of 75% (vol/vol) EtOH. These samples were loaded to HPLC in order to measure the concentration of alanine, glutamic acid, and diaminopimelic acid.

### Porcine Intestinal Epithelial Cells

The PIE cell line was originally derived from intestinal epithelia isolated from an unsuckled neonatal swine (16). PIE cells are intestinal non-transformed cultured cells that assume a monolayer with a cobblestone and epithelial-like morphology and with close contact between cells during culture (16–18). PIE cells were maintained in Dulbecco’s modified Eagle’s medium (DMEM) (Invitrogen Corporation, Carlsbad, CA) supplemented with 10% fetal calf serum (FCS), 100 mg/mL streptomycin and 100 U/mL penicillin at 37°C in an atmosphere of 5% CO_2_ (9, 10, 18, 19).

### Immunomodulatory Effect of Lactobacilli in Porcine Intestinal Epithelial Cells

The study of the immunomodulatory capacity of *L. plantarum* WT and *L. plantarum* Δ*dltD* was performed in PIE cells as described previously (9, 10). PIE cells were seeded at 3 × 10^4^ cells per well in 12-well type I collagen-coated plates (Sumitomo Bakelite Co., Tokyo, Japan) and cultured for 3 days. After changing medium, lactobacilli (5 × 10^8^ cells/ml) were added, and 48 h later, each well was washed vigorously with medium at least three times to eliminate all stimulants. Then, cells were stimulated with poly(I:C) (60 μg/ml) for 12 h for RT-PCR studies.

### Quantitative Expression Analysis by Two-Step Real-Time Quantitative PCR

Two-step real-time quantitative PCR (qPCR) was performed to characterize the expression of selected genes in PIE cells as described previously (9). TRIzol reagent (Invitrogen) was used for total RNA isolation from each PIE cell sample, and Quantitect reverse transcription (RT) kit (Qiagen, Tokyo, Japan) was used for the synthesis of all cDNAs according to the manufacturer’s recommendations. qPCR was carried out using a 7300 real-time PCR system (Applied Biosystems, Warrington, United Kingdom) and the Platinum SYBR green qPCR SuperMix uracil-DNA glycosylase (UDG) with 6-carboxyl-X-rhodamine (ROX) (Invitrogen). The primers used in this study were described before (9, 10, 20). The PCR cycling conditions were 2 min at 50°C, followed by 2 min at 95°C, and then 40 cycles of 15 s at 95°C, 30 s at 60°C, and 30 s at 72°C. The reaction mixtures contained 5 μl of sample cDNA and 15 μl of master mix, which included the sense and antisense primers. According to the minimum information for publication of quantitative real-time PCR experiments guidelines, β-actin was used as a housekeeping gene because of its high stability across porcine various tissues (17, 20, 21). Expression of β-actin was used to normalize cDNA levels for differences in total cDNA levels in the samples.

### Animals, Feeding Procedures, and Poly(I:C) Challenge

Male 6-week-old BALB/c mice were obtained from the closed colony kept at CERELA-CONICET (Tucuman, Argentina). Animals were housed in individual plastic cages in a controlled atmosphere (22°C ± 2°C temperature, 55% ± 2% humidity) with a 12-h light/dark cycle. *L. plantarum* WT and *L. plantarum* Δ*dltD* were orally administered to different groups of mice for five consecutive days at a dose of 10^8^ cells/mouse/day in a controlled volume of the drinking water supplemented with non-fat milk (10%) to ensure the complete consumption of the viable bacteria (22, 23). The treated groups and the untreated control mice were fed a conventional balanced diet *ad libitum*. Mice were challenged by the intraperitoneal route with 100 μl of PBS containing 30 μg poly(I:C) according to our previous publication (11). Biochemical markers of injury as well as intestinal cytokine concentrations were evaluated 2 days after poly(I:C) administration as described below. All experiments were carried out in compliance with the Guide for Care and Use of Laboratory Animals and approved by the Ethical Committee of Animal Care at CERELA, Argentina (protocol number BIOT-CRL/14) (11).

### Biochemical Markers of Injury

Lactate dehydrogenase (LDH) and aspartate aminotransferase (AST) activities were determined in the serum to evaluate general toxicity of poly(I:C) in mice challenged by the intraperitoneal injection. Blood samples were obtained through cardiac puncture under anesthesia. LDH and AST activities, expressed as units per liter of serum, were determined by measuring the formation of the reduced form of nicotinamide adenine dinucleotide (NAD) using the Wiener reagents and procedures (Wiener Lab, Buenos Aires, Argentina) (11).

### Cytokine Concentrations

Serum samples were obtained as described before. Intestinal fluid samples were obtained according to our previous publication (11). Briefly, the small intestine was flushed with 5 ml of PBS and the fluid was centrifuged (10,000? × *g*, 4°C 10?min) to separate particulate material. The supernatant was kept frozen until use.

Tumor necrosis factor (TNF)-α, IL-6, IL-10, IL-15, IFN-β, and IFN-γ concentrations in serum and intestinal fluid samples were measured with commercially available enzyme-linked immunosorbent assay (ELISA) technique kits following the manufacturer’s recommendations (R&D Systems, MN, United States).

### Total and Differential Blood Leukocyte Counts

The total number of leukocytes was determined with a hemocytometer. Differential cell counts were performed by counting 200 cells in blood smears stained with May Grünwald Giemsa stain using a light microscope (1,000×), and absolute cell numbers were calculated (24).

### Intestinal Intraepithelial Lymphocytes

Intraepithelial lymphocytes were isolated according to our previous publication (11). Briefly, Peyer’s patches were excised; the small intestine was opened longitudinally and cut into 5-mm-long pieces. Samples were washed twice in PBS containing 150 μg/ml streptomycin and 120 U/ml penicillin. The pieces were then stirred at 37°C in prewarmed RPMI 1640 containing 150 μg/ml streptomycin, 120 U/ml penicillin, and 5% FCS for 30 min, followed by vigorous shaking for 40 s. This process was repeated, and the supernatants were passed through a small cotton-glass wool column to remove cell debris and were then separated on a Percoll density gradient (Amersham Biosciences). A discontinuous density gradient (40% and 70%) was used. The cells that layered between the 40% and 70% fractions were collected as IELs. These IELs contained >90% CD3^+^ cells, as determined by fluorescence-activated cell sorting (FACS) analysis.

Cellular phenotypes in IEL populations were analyzed by flow cytometry using fluorescein isothiocyanate (FITC)-conjugated anti-CD3 and phycoerythrin (PE)-conjugated anti-NK1.1 (PK136) and anti-CD8α (CTCD8b) (R&D Systems). Anti-NKG2D (CX5) was purchased from eBioscience (San Diego, CA, United States). To prevent non-specific binding, respective isotype Abs were used as controls. Images of labeled cells were acquired on a BD FACSCalibur^TM^ flow cytometer (BD Biosciences) and analyzed with FlowJo software (TreeStar).

### Statistical Analysis

Experiments were performed in triplicate, and results were expressed as mean ± standard deviation (SD). After verification of the normal distribution of data, two-way ANOVA was used. Tukey’s test (for pairwise comparisons of the means) was used to test for differences between the groups. Differences were considered significant at *P* < 0.05 or *P* < 0.01.

## Results

### Development of *L. plantarum* Δ*dltD*

*Lactobacillus plantarum* Δ*dltD* was produced by double-crossover (Figure 1A), and the deletion of 27-bp in the *dltD* gene was confirmed by PCR analysis (Figure 1B) and sequencing (data not shown). It has been reported that LTA mutations in lactobacilli can lead to modifications on growth, cell wall organization, cell morphology, or cell division. The Δ*dltD* mutant from *Lactobacillus rhamnosus* GG had defects in septum formation and an increased cell length (25), while the Δ*dltD* mutant from *L. plantarum* WCFS1 showed increased autolysis, defects in cell separation, and enhanced cell length (26). Then, the ability of the mutant strain *L. plantarum* Δ*dltD* to grow in MRS medium as well as its surface and shape characteristics were evaluated by comparing it with the WT *L. plantarum* CRL1506. As shown in Figures 1C,D, the deletion of 27 bp in the *dltD* gene did not affect the ability of *L. plantarum* Δ*dltD* to grow neither altered its shape or surface characteristics when compared to *L. plantarum* WT. Those results are in line with the findings of (27) that showed that the Δ*dltD* mutant from *L. acidophilus* NCFM had no growth or morphological defects.

**FIGURE 2:**
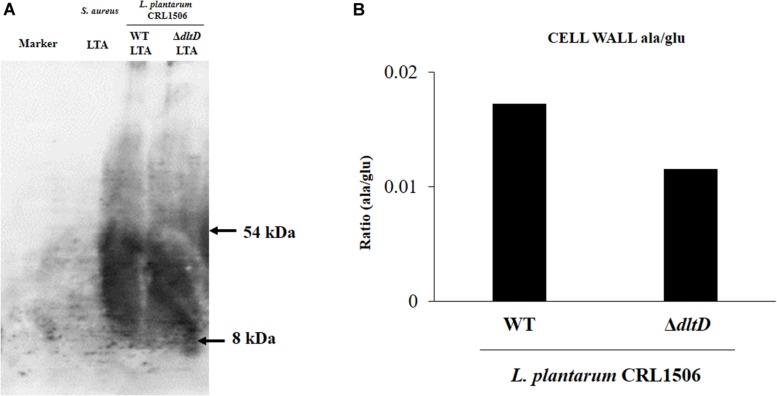
Development of a D-alanyl-lipoteichoic acid biosynthesis protein (*dltD*) mutant from *Lactobacillus plantarum* CRL1506. Analysis of lipoteichoic acid (LTA) by **(A)** Western blot and **(B)** high-performance liquid chromatography (HPLC) (Ala/Glu ratio) in wild-type (WT) and Δ*dltD* mutant from *Lactobacillus rhamnosus* CRL1506.

In addition, LTA in WT and Δ*dltD* strains was analyzed by Western blot and HPLC. A reduction in LTA content (Figure 2A) as well as a decrease in the Ala/Glu ratio (Figure 2B) were found in *L. plantarum* Δ*dltD* when compared with the WT strain.

### Effect of *L. plantarum*Δ*dltD* in Toll-Like Receptor 3-Challenged Porcine Intestinal Epithelial Cells

**FIGURE 3:**
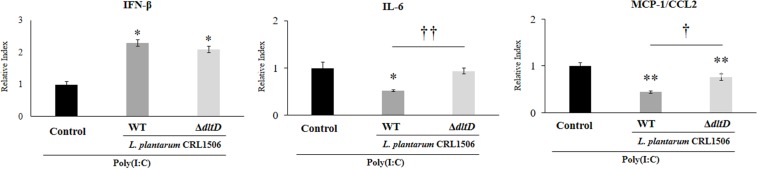
Effect of a D-alanyl-lipoteichoic acid biosynthesis protein (*dltD*) mutant from *Lactobacillus plantarum* CRL1506 on innate antiviral immune response triggered by Toll-like receptor 3 (TLR3) activation in porcine intestinal epithelial (PIE) cells. Expression of interferon (*IFN*)-β, interleukin (*IL*)*-6*, and monocyte chemoattractant protein (*MCP*)*-1/CCL2* genes in PIE cells treated with wild-type (WT) or Δ*dltD* mutant from *Lactobacillus rhamnosus* CRL1506 and challenged with the viral molecular associated pattern poly(I:C). PIE cells with no lactobacilli treatment and stimulated with poly(I:C) were used as controls. The results represent data from three independent experiments. Significant differences when compared to the control group: *P < 0.05, **P < 0.01. Significant differences when compared to the indicated group: ^†^*P* < 0.05, ^†⁣†^*P* < 0.01.

In order to evaluate whether the alteration of the LTA in *L. plantarum* CRL1506 induced modifications on its immunomodulatory activities, we first studied *in vitro* its ability to functionally modulate immune factor expression in PIE cells after the activation of TLR3. For this purpose, PIE cells were treated with *L. plantarum*Δ*dltD* or *L. plantarum* WT and then challenged with the TLR3 agonist poly(I:C). The expressions of *IFN-*β, *IL-6*, and *CCL2* were evaluated by qPCR (Figure 3). As it was described previously (9, 10), the treatment of PIE cells with *L. plantarum* WT significantly increased the expression of IFN-β, while a decrease in the mRNA levels of *IL-6* and *CCL2* was observed. No differences between Δ*dltD* and WT strains were found when the expression of *IFN-*β was compared. However, the expression of both *IL-6* and *CCL2* were significantly higher in Δ*dltD-*treated PIE cells when compared to those stimulated with *L. plantarum* WT (Figure 3).

### Effect of *L. plantarum*Δ*dltD* in Toll-Like Receptor 3-Induced Inflammatory Damage in Mice

**FIGURE 4:**
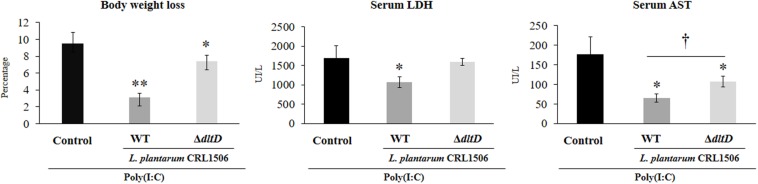
Effect of a D-alanyl-lipoteichoic acid biosynthesis protein (*dltD*) mutant from *Lactobacillus plantarum* CRL1506 on the inflammatory damage induced by Toll-like receptor 3 (TLR3) activation in mice intestine. Body weight loss and serum biochemical markers lactate dehydrogenase (LDH) and aspartate aminotransferase (AST) in mice orally treated with wild-type (WT) or Δ*dltD* mutant from *L. rhamnosus* CRL1506 and challenged with an intraperitoneal injection of the viral molecular associated pattern poly(I:C). Mice with no lactobacilli treatment and challenged with poly(I:C) were used as controls. The results represent data from three independent experiments. Significant differences when compared to the control group: *P < 0.05, **P < 0.01. Significant differences when compared to the indicated group: ^†^*P* < 0.05.

Taking into consideration that the *in vitro* studies suggested that the alteration of LTA in *L. plantarum* CRL1506 would also alter its anti-inflammatory properties, we next aimed to evaluate whether *L. plantarum*Δ*dltD* was able to modulate the intestinal inflammatory response and protect against the damage induced by TLR3 activation by using an *in vivo* model. Then, different groups of mice were fed *L. plantarum*Δ*dltD* or *L. plantarum* WT and then challenged with the TLR3 agonist poly(I:C) as described in *Materials and Methods*. We evaluated body weight loss and the biochemical markers LDH and AST in serum to study the general health status of mice after poly(I:C) administration (Figure 4). As it was reported previously (11), poly(I:C) challenge significantly decreased the body weight gain and increased the levels of serum LDH and AST. Mice treated with *L. plantarum* WT showed reduced levels of injury biochemical markers and improved body weight gain when compared to control mice (Figure 4). On the other hand, mice treated with *L. plantarum*Δ*dltD* showed levels of serum LDH that were not different from those observed in the control group. In addition, the mutant strain was able to reduce the alteration of body weight gain and the levels of serum AST, but its effect was significantly lower when compared to *L. plantarum* WT (Figure 4).

**FIGURE 5:**
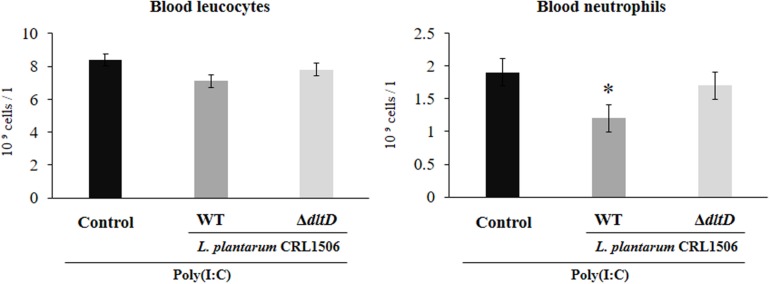
Effect of a D-alanyl-lipoteichoic acid biosynthesis protein (*dltD*) mutant from *Lactobacillus plantarum* CRL1506 on the inflammatory response induced by Toll-like receptor 3 (TLR3) activation in mice intestine. Blood leukocyte and neutrophil counts in mice orally treated with wild-type (WT) or Δ*dltD* mutant from *Lactobacillus rhamnosus* CRL1506 and challenged with an intraperitoneal injection of the viral molecular associated pattern poly(I:C). Mice with no lactobacilli treatment and challenged with poly(I:C) were used as controls. The results represent data from three independent experiments. Significant differences when compared to the control group: *P < 0.05.

Total and differential blood leukocyte counts were also evaluated to study the systemic inflammatory response. Challenge with poly(I:C) significantly increased the number of leukocytes and neutrophils in blood. However, the numbers of neutrophils were lower in *L. plantarum* WT-treated mice when compared to the *L. plantarum*Δ*dltD* and control groups (Figure 5).

### Effect of *L. plantarum*Δ*dltD* on the Innate Immune Response Induced by Toll-Like Receptor 3

**FIGURE 6:**
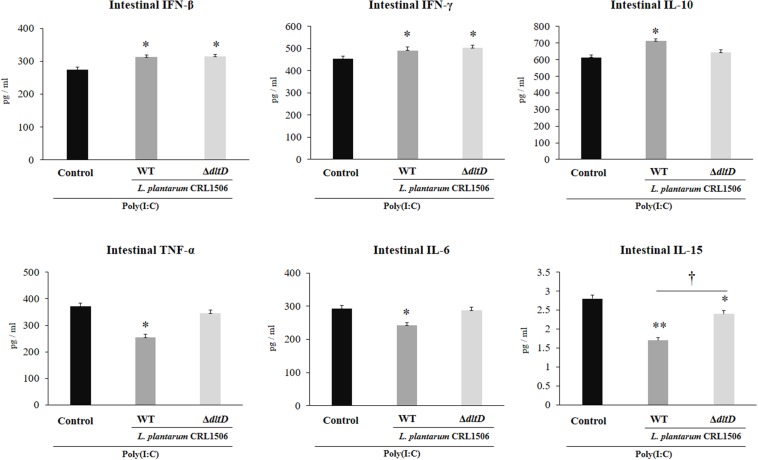
Effect of a D-alanyl-lipoteichoic acid biosynthesis protein (*dltD*) mutant from *Lactobacillus plantarum* CRL1506 on the innate antiviral immune response triggered by Toll-like receptor 3 (TLR3) activation in mice intestine. Levels of intestinal interferon (IFN)-β, IFN-γ, interleukin (IL)-10, tumor necrosis factor (TNF)-α, IL-6, and IL-15 in mice orally treated with wild-type (WT) or Δ*dltD* mutant from *Lactobacillus rhamnosus* CRL1506 and challenged with an intraperitoneal injection of the viral molecular associated pattern poly(I:C). Mice with no lactobacilli treatment and challenged with poly(I:C) were used as controls. The results represent data from three independent experiments. Significant differences when compared to the control group: *P < 0.05, **P < 0.01. Significant differences when compared to the indicated group: ^†^*P* < 0.05.

**FIGURE 7:**
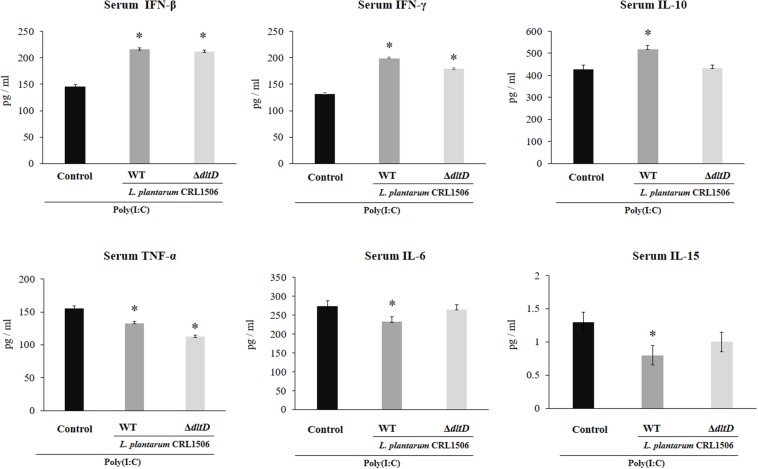
Effect of a D-alanyl-lipoteichoic acid biosynthesis protein (*dltD*) mutant from *Lactobacillus plantarum* CRL1506 on the innate antiviral immune response triggered by Toll-like receptor 3 (TLR3) activation in mice intestine. Levels of serum interferon (IFN)-β, IFN-γ, interleukin (IL)-10, tumor necrosis factor (TNF)-α, IL-6, and IL-15 in mice orally treated with wild-type (WT) or Δ*dltD* mutant from *Lactobacillus rhamnosus* CRL1506 and challenged with an intraperitoneal injection of the viral molecular associated pattern poly(I:C). Mice with no lactobacilli treatment and challenged with poly(I:C) were used as controls. The results represent data from three independent experiments. Significant differences when compared to the control group: *P < 0.05.

The intraperitoneal administration of poly(I:C) significantly increased the levels of pro-inflammatory cytokines in the intestine (Figure 6) and serum (Figure 7). Both *L. plantarum*Δ*dltD* and *L. plantarum* WT were able to improve the production of the antiviral factors IFN-β and IFN-γ in the intestine and serum when compared to control mice and with no differences between lactobacilli. As reported previously (11), *L. plantarum* WT significantly reduced the concentrations of TNF-α, IL-6, and IL-15 in intestinal fluid (Figure 6) and serum (Figure 7). *L. plantarum*Δ*dltD* was as effective as the WT strain to diminish the concentrations of serum TNF-α (Figure 7). In addition, *L. plantarum*Δ*dltD* was able to reduce the levels of intestinal IL-15, but it was not efficient as the WT strain (Figure 6). The levels of intestinal TNF-α and IL-6 (Figure 6) as well as serum IL-6 and IL-15 (Figure 7) in Δ*dltD-*treated mice were not different from controls.

Only *L. plantarum* WT treatment was able to significantly increase the levels of intestinal and serum IL-10, while the concentrations of this regulatory cytokine in *L. plantarum*Δ*dltD-*treated mice were not different from controls (Figures 6, 7).

**FIGURE 8:**
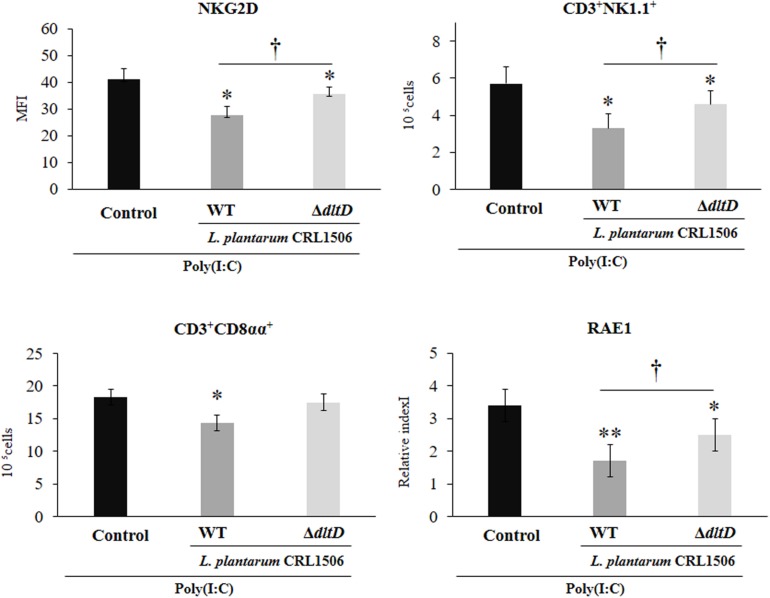
Effect of a D-alanyl-lipoteichoic acid biosynthesis protein (*dltD*) mutant from *Lactobacillus plantarum* CRL1506 on the innate antiviral immune response triggered by Toll-like receptor 3 (TLR3) activation in mice intestine. Numbers of CD3^+^NK1.1^+^ and CD3^+^CD8αα^+^ intraepithelial lymphocytes (IELs), expression of NKG2D on IELs, and expression of intestinal *RAE1* gene in mice orally treated with wild-type (WT) or Δ*dltD* mutant from *Lactobacillus rhamnosus* CRL1506 and challenged with an intraperitoneal injection of the viral molecular associated pattern poly(I:C). Mice with no lactobacilli treatment and challenged with poly(I:C) were used as controls. The results represent data from three independent experiments. Significant differences when compared to the control group: *P < 0.05, **P < 0.01. Significant differences when compared to the indicated group: ^†^*P* < 0.05.

Finally, we assessed the changes in the populations of intestinal IELs in mice challenged with the TLR3 agonist poly(I:C). For this purpose, we studied variations in CD3^+^NK1.1^+^ and CD3^+^CD8αα^+^ populations as well as NKG2D expression within IELs by flow cytometry. Poly(I:C) administration induced an increase in all these three parameters evaluated (Figure 8). *L. plantarum* WT-treated mice showed significantly decreased numbers of intestinal CD3^+^NK1.1^+^ and CD3^+^CD8αα^+^ cells and NKG2D expression when compared to controls. On the contrary, the treatment of mice with *L. plantarum*Δ*dltD* was not able to reduce the numbers of CD3^+^CD8αα^+^ cells (Figure 8). The Δ*dltD* strain reduced the numbers of CD3^+^NK1.1^+^ cells and the expression of NKG2D, but those parameters were significantly higher than those observed in WT-treated mice (Figure 8). We also evaluated the expression of the NKG2D ligand RAE1 by qPCR. TLR3 activation increased the expression of RAE1 in all the experimental groups (Figure 8). Both *L. plantarum*Δ*dltD* and *L. plantarum* WT were able to reduce RAE1 expression in the intestinal tissue of mice; however, the levels of expression in WT-treated mice were significantly lower than those observed in Δ*dltD*-treated animals (Figure 8).

## Discussion

Research from the last decade investigating the immunobiotic effector molecules has demonstrated that each individual immunobiotic strain has a characteristic set of molecules, which are responsible of its interaction with the host’s immune receptors (28–30). Surface molecules of immunobiotic microorganisms, including exopolysaccharides, cell wall peptidoglycan, and LTA, have been frequently associated with their immunomodulatory effects (28, 29, 31, 32). In this regard, several reports have emphasized the importance of LTA in the immunomodulatory properties of some probiotic lactobacilli.

A Δ*dltD* mutant from *L. plantarum* WCFS1, affected in the LTA biosynthesis pathway, was found to incorporate much less D-Ala in its LTA than the WT strain (33). The D-Ala content defect in the LTA significantly altered the immunomodulatory ability of the Δ*dltD* mutant from WCFS1 strain. The mutant showed a higher anti-inflammatory activity when compared to the parental strain in both *in vitro* and *in vivo* studies. The Δ*dltD* mutant from *L. plantarum* WCFS1 was more efficient than the WT strain to improve IL-10 production and reduce TNF-α, IL-12, and IFN-γ in human PBMCs and monocytes. In addition, the treatment of mice with the Δ*dltD* mutant from *L. plantarum* WCFS1 significantly reduced the percentage of mice displaying diarrhea after trinitrobenzene sulfonic acid (TNBS) administration when compared with animals receiving the *L. plantarum* WT strain (33). Other reports provided similar results for *L. acidophilus* NCFM (27) and *L. rhamnosus* GG (34) in which LTA mutant strains improved the production of anti-inflammatory cytokines in immune cells and reduced the severity of dextran sulfate sodium (DSS)-induced colitis in mice. Those results indicated that LTA from some lactobacilli strains have immune stimulatory effects. In line with this assumption, it was demonstrated that LTA from *L. fermentum* YIT0159 and *L. casei* YIT9029 induces TNF-α expression in macrophages (35).

In contrast to the pro-inflammatory properties of lactobacilli LTA, others works reported that those molecules have anti-inflammatory activities in some *Lactobacillus* strains. The LTA from *L. plantarum* K8 was a weaker inducer of nitric oxide (NO) and TNF-α in macrophages when compared with the LTA from *Bacillus subtilis* or *Staphylococcus aureus* (36). The LTA from the K8 strain was able to act as an antagonist in the induction of TNF-α by monocytes challenged with lipopolysaccharide (LPS) (37), peptidoglycan of *Shigella flexneri* (38), or LTA from *S. aureus* (39). Moreover, the purified LTA from *L. plantarum* K8 differentially modulated the response of human keratinocytes to the stimulation with TNF-α or IFN-γ (40). LTA-treated keratinocytes had a significantly reduced activation of TNF-α/p65/p38 and IFN-γ/STAT1/2/JAK2 pathways with a subsequent reduced expression of inflammatory factors such as the complement protein C3. It was also described that the LTA from *L. plantarum* K8 had the ability to regulate the TLR2-triggered inflammatory response in intestinal epithelial cells (IECs). Caco-2 cells treated with *L. plantarum* K8 LTA and then challenged with the TLR2 agonist Pam2CSK4 showed significantly lower expression of IL-8 when compared to control cells (41). *In vivo* studies also support the anti-inflammatory activities of LTA from lactobacilli. *L. casei* BL23 had been shown to exert beneficial effects in the DSS-induced murine model of ulcerative colitis, and it was reported that the Δ*dltD* mutant from the BL23 strain was unable to protect against the inflammatory damage induced by DSS (42).

These contrasting results could be explained by the chemical and structural differences of LTA molecules. Chemical and molecular studies of LTA highlighted the variety in their structure by means of the number of acyl chains and the degree of saturation in the lipid chain as well as the D-ala or sugars substitutions on the Gro-P chains (43) that could explain their differential immunomodulatory effects. In addition, it should be considered that the immunomodulatory activities of LTA derived from lactobacilli have been studied in different contexts of cytokine- or bacteria-induced inflammatory responses, adding additional complexity to the interpretations. Of note, the potential beneficial effect of LTA from lactobacilli in the context of viral infection or viral pattern recognition receptors-mediated inflammation has not been explored in depth.

In an interesting study performed by Kim et al. (44), it was demonstrated that the LTA isolated from *L. plantarum* K8 was able to regulate mitogen-activated protein kinase (MAPK) phosphorylation and nuclear factor (NF)-κB activation and reduce IL-8 production in IPEC-J2 cells in response to the challenge with the TLR3 agonist poly(I:C). The work also demonstrated that LTA isolated from other lactobacilli strains including *L. delbrueckii*, *L. sakei*, and *L. rhamnosus* GG did not have the ability to differentially modulate IL-8 secretion in poly(I:C)-challenged IECs. Moreover, comparative studies evaluating the immunomodulatory potential of naive LTA and dealanylated LTA from *L. plantarum* K8 clearly showed that the dealanylated LTA failed to inhibit IL-8 secretion in IECs after the activation of TLR3. In line with these previous results, we demonstrated in this work that the LTA is involved in the ability of the immunobiotic strain *L. plantarum* CRL1506 to reduce the expression of pro-inflammatory factors in IECs challenged with poly(I:C). Previously, we demonstrated that PIE cells are able to upregulate the expression of several inflammatory factors including *TNF-*α, *IL-6* (9, 10), *IL-1*β, *IL-15*, and the chemokines *CCL4*, *CXCL2*, *CXCL5*, *CCL8*, *CXCL10*, and *CCL5* (9) in response to poly(I:C) challenge. We also reported that PIE cells treated with *L. plantarum* CRL1506 prior to TLR3 activation had a reduced expression of inflammatory cytokines and chemokines (9, 10). Here, by using the expression of *IL-6* and *CCL2* as markers of inflammation, we demonstrated that the alteration in the incorporation of D-ala in the LTA molecule of *L. plantarum* CRL1506 significantly diminished the ability of the immunobiotic strain to modulate inflammatory factor expression.

**FIGURE 9:**
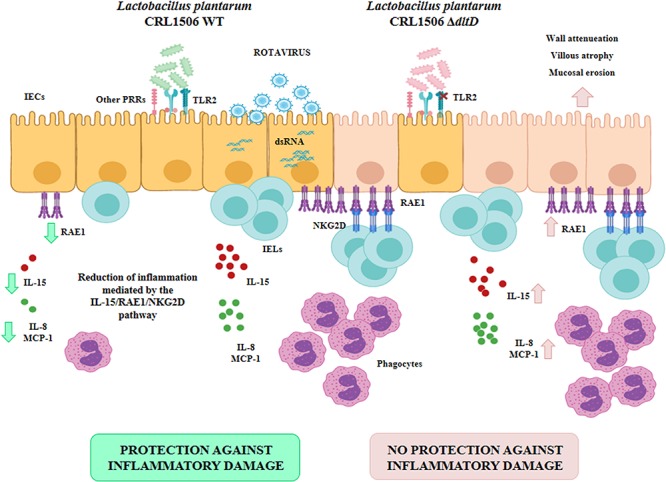
Role of lipoteichoic acid in the beneficial effects *Lactobacillus plantarum* CRL1506 on the innate antiviral immune response triggered by Toll-like receptor 3 (TLR3) activation in the intestine.

Moreover, this work is the first in demonstrating in an *in vivo* model the role of LTA from an immunobiotic strain in the beneficial modulation of the intestinal innate antiviral immune response triggered by TLR3 activation. We demonstrated previously that *L. plantarum* CRL1506 reduced the TLR3-induced small intestinal injury in mice by regulating the production of pro-inflammatory cytokines and the interaction of IECs with IELs (11) (Figure 9). Cell–cell interaction between IECs and IELs is essential as a first line of defense against viruses (45–47). In the context of viral infection, the cell death program of IECs is regulated by their upregulation in the expression of IL-15, which is a cytokine capable of stimulating the activation of CD3^+^NK1.1^+^ IELs and inducing perforin-mediated killing of virus-infected epithelial cells (46). The activation of TLR3 in the intestinal tract of mice enhances the expression of RAE1 in IECs, allowing their destruction by interacting with NKG2D expressed on CD3^+^NK1.1^+^ IELs (48). This mechanism of small intestinal injury that induces villous atrophy and mucosal erosion (49) is particularly relevant for rotavirus infection since it was reported that treatment of mice with poly(I:C) or purified dsRNA from rotavirus increased intestinal injury in a CD8αα^+^NKG2D^+^- and RAE1-dependent manner (48, 49). In fact, blockade of RAE1-NKG2D interaction avoids the cytotoxic effect of IELs on IECs and prevents acute small intestinal injury in mice challenged with rotavirus genomic dsRNA (48). Of interest, the oral administration of *L. plantarum* CRL1506 to mice prior the intraperitoneal challenge with poly(I:C) significantly reduced the inflammatory-mediated intestinal tissue damage through the downregulation of TNF-α, IL-1β, and IL-8, particularly IL-15 and RAE1, and the reduction of the numbers and activation (NKG2D^+^ expression) of CD3^+^NK1.1^+^CD8αα^+^ cells (11). Here, we observed that *L. plantarum*Δ*dltD* had a reduced capacity for the protection of mice against the TLR3-mediated inflammatory damage as demonstrated by the analysis of body weight loss and serum markers of damage. Moreover, we observed that the Δ*dltD* mutant from *L. plantarum* CRL1506 was inefficient for reducing the expression of IL-15 and RAE1 in IECs or the expression of NKG2D in IELs (Figure 9), indicating that the LTA from this immunobiotic strain is the main molecule involved in their ability to regulate IL-15/RAE1/NKG2D death pathway.

It was reported that the expression of NKG2D ligands such as RAE1 on IECs is strongly modulated by the intestinal microbiota. Studies in germ-free mice and ampicillin-treated mice lacking gut commensal microbiota demonstrated that the absence of microbial stimuli significantly upregulates the expression of NKG2D ligands in IECs (50). Then, taking into consideration the results of this work, it is tempting to speculate that LTA would be at least one of the molecules used by the gut microbiota to regulate the innate antiviral immune response in the intestinal tract. In support of this assumption, it was shown that gut commensal bacteria help in the establishment of a regulatory milieu in the intestinal mucosa by increasing the expression of IL-10 and TGF-β, which diminish the expression of NKG2D ligands on the surface of IECs (51, 52). Of note, *L. plantarum* CRL1506 is able to improve the intestinal levels of IL-10 in poly(I:C)-challenged mice, while the Δ*dltD* mutant from CRL1506 strain was inefficient for inducing this effect. The molecular mechanisms and specific importance of LTA from microbiota and immunobiotic strains in the protection against viral inflammatory-mediated intestinal damage require further research.

Interestingly, although the *L. plantarum* CRL1506 Δ*dltD* was less efficient in regulating the TLR3-mediated inflammatory response in both *in vitro* and *in vivo* experiments, the mutant strain was as efficient as the WT to improve the levels of IFN-β in the intestine. It was reported that the LTA of probiotic bacteria mediates its immunomodulatory activities in a TLR2-dependent manner (33, 37, 39). Our previous studies evaluating the interaction of *L. plantarum* CRL1506 with IECs demonstrated that TLR2 is involved in the ability of the strain to differentially modulate cytokines in response to TLR3 activation (10). The use of anti-TLR2 Abs inhibited the ability of the CRL1506 strain to modulate *TNF-*α and *IL-6* transcripts in PIE cells. However, when blocking anti-TLR2 Abs were used to evaluate the influence of *L. plantarum* CRL1506 on the production of IFN-β in PIE cells, it was evident that this receptor was not involved in the modulation of this antiviral factor (10). Those previous findings and the results presented here indicate that TLR2 should be linked to the effect of biologically active LTA on IECs, but that other host receptors and microbial molecules might also be involved in *L. plantarum* CRL1506 recognition by the intestinal epithelium, and in its beneficial influence on intestinal innate antiviral immune response. In addition, our results demonstrated that *L. plantarum* CRL1506 Δ*dltD* was as effective as the WT to reduce the serum levels of AST and TNF-α and improve serum IFN-γ in poly(I:C)-challenged mice. It have been proposed that the systemic effects of orally administered probiotics are mediated mainly by the interaction of these beneficial microbes with innate immune cells such as macrophages. In the analyses of the profiles of cytokines induced by probiotic lactobacilli, it was observed that changes in serum cytokines reflected their production by intestinal and peritoneal macrophages (53, 54). Therefore, it is tempting to speculate that both Δ*dltD* and WT strains from *L. plantarum* CRL1506 would have the same capacity to modulate macrophage activity and that the TRL2-LTA interaction would not be involved in such modulation. More detailed experiments evaluating the interaction of both Δ*dltD* and WT strains with macrophages would be of great importance to improve our understanding of the role of LTA in the immunomodulatory activity of *L. plantarum* CRL1506.

In conclusion, although further studies are needed to elucidate all the active bacterial components responsible for the immunomodulatory capacities of *L. plantarum* CRL1506, the results of this work indicate that LTA is an important molecule involved in the immunobiotic effects induced by this strain in the intestinal innate antiviral immune response triggered by TLR3 activation. In line with previous studies (27, 33, 34), our results also indicate that the use of immunobiotic mutants carrying directed mutations could help to improve the understanding of the molecular interactions of immunobiotics with the host immune system and how that interaction beneficially modulates the innate antiviral immune response. This study is an important step toward insights in the mode of action of immunobiotics and their influence in the protection against viral-mediated inflammation.

## Data Availability Statement

The datasets generated for this study are available on request to the corresponding author.

## Ethics Statement

The animal study was reviewed and approved by the Ethical Committee of Animal Care at CERELA, Argentina.

## Author Contributions

JV, YS, and HK designed the study. JV and HK contributed to the manuscript writing. HM, LoA, KT, LeA, and RF did the laboratory work. MI and LeA did the statistical analysis. MV-P, HT, YS, HK, and JV contributed to the data analysis and interpretation. All authors read and approved the manuscript.

## Conflict of Interest

The authors declare that the research was conducted in the absence of any commercial or financial relationships that could be construed as a potential conflict of interest.
